# Benefits of negative pressure wound therapy with fat migration during revisional total hip arthroplasty in an obese patient: A case report

**DOI:** 10.1097/MD.0000000000036726

**Published:** 2023-12-29

**Authors:** Zoya Morani, Aashna Mehta, Fnu Parul, Helen Huang, Mohammad Raza Khan, Nialish Javaid, Rachael Suzanne Ninan, Andrew Awuah Wireko, Luqman Muhammed, Fatima Aman Siddiqui

**Affiliations:** a Washington University of Health and Science, San Pedro, Belize; b University of Debrecen-Faculty of Medicine, Debrecen, Hungary; c Pandit Bhagwat Dayal Sharma Post Graduate Institute of Medical Sciences, Rohtak, India; d Royal College of Surgeons in Ireland, University of Medicine and Health Science, Dublin, Ireland; e Humboldt Park Health, Chicago, IL, USA; f Windsor University School of Medicine, Cayon, St Kitts and Nevis; g Sumy State University, Sumy, Ukraine.

**Keywords:** negative pressure wound therapy, obesity, orthopedic surgery, revisional arthroplasty, total hip replacement

## Abstract

**Introduction:**

A 38-year-old African American woman presented with right hip pain and movement restriction. Her medical history included a right hip and knee arthroplasty 10 years prior, history of Slipped Capital Femoral Epiphysis and osteonecrosis of the hip. Preoperative assessment was significant for multiple comorbidities such as obesity (BMI > 38), hypertension, asthma.

**Patient concerns:**

The patient presented with right hip pain, rated 7/10, and restricted hip flexion, adduction, and abduction.

**Diagnosis:**

Recent imaging showed eccentric deterioration of the polyethylene lining of her prosthesis, acetabular hypertrophy on her right hip prosthesis, and chronic deformity of the pubic bone.

**Interventions:**

Based on these findings, a revisional total hip arthroplasty was performed. After the surgical procedure, the WoundVAC and the percutaneous drain were applied outside the tensor fascia lata to reduce seroma and hematoma formation. Postoperative pain control, antibiotics and DVT prophylaxis were given. On post-op day 3, an irrigation and debridement with delayed primary wound closure was performed under sterile conditions.

**Outcomes:**

On postoperative assessment, the wound demonstrated adequate healing without any signs of infection. Sutures and staples were removed 4 weeks post-op. Upon palpation there was no edema, effusions, temperature changes, tenderness. Clinical inspection revealed symmetrical alignment of the pelvis and hips. Range of motion testing revealed restriction beyond 80 degrees upon flexion and beyond 5 degrees of adduction and 10 degrees of abduction. The surgical site was noted to be healed at 6 weeks post-op. The patient continued to do well to date, without exacerbations.

**Conclusion:**

Obesity increases the risk of post-operative complications and wound healing failure. Therefore, Vacuum-assisted wound closure (WoundVAC), a type of negative pressure wound therapy, was applied outside the tensor fascia lata post-operatively, where the surgical incision was made. Negative pressure wound therapy facilitates wound healing by stimulating angiogenesis and promoting granulation tissue formation, which in turn can reduce the risk of surgical site infection in obese patients undergoing total hip arthroplasty. Highlighted is the mechanism of fat migration in the promotion of wound healing after preoperative weight loss and exercise.

## 1. Introduction

Total hip arthroplasty (THA) is one of the most common orthopedic procedures performed in the United States, with over 450,000 procedures performed annually. Recent studies have demonstrated a high prevalence of THAs in obese patients, linking a high BMI to post-operative wound healing complications.^[1]^ Delayed wound healing in patients with comorbidities, especially during invasive procedures, can result in increased risk for pain, morbidity and additional reconstructive surgeries.^[2]^ However, novel wound closure techniques, such as negative pressure wound therapy (NPWT) can reduce readmission rate and improve wound healing in obese patients undergoing orthopedic procedures.^[3]^ Vacuum-assisted wound closure (WoundVAC), is a type of NPWT in which the wound is sealed with foam dressing, with a drainage tube that connects the wound to a portable vacuum pump. The benefits of NPWT include reduced edema, improved blood circulation, angiogenesis, tissue perfusion, granulation tissue formation, and increased bacterial clearance.^[3,4]^ Although the application of NPWT has been previously discussed, the concept of fat migration is promising yet unexplored. This case report highlights the benefits of using NPWT for wound closure in an obese patient undergoing revisional THA and discusses the role of fat migration in facilitating wound healing.

This case report has been reported in line with the SCARE Criteria.^[5]^

## 2. Case presentation

A 38-year-old, African-American female presented with right hip pain, rated 7/10, and restricted hip flexion, adduction and abduction. Her history included childhood Slipped Capital Femoral Epiphysis and osteonecrosis with comorbidities such as obesity (BMI 38), hypertension and asthma, managed with nifedipine and albuterol respectively. The patient also has a past medical history of Hepatitis C. She has a surgical history of right hip replacement, a c-section and cholecystectomy. The patient was allergic to sumatriptan. The patient reported a family history of Diabetes in her maternal grandmother and pulmonary embolism in her mother who passed away at the age of 38. The patient denies smoking and consumes wine occasionally. The patient underwent a right hip arthroplasty with an arthroscopy of the knee 10 years earlier. Upon review of systems, the patient reported additional problems of, constipation, heartburn, joint pain and back problems. Recent imaging revealed eccentric wear of the polyethylene liner of her right hip prosthesis, with cyst formation as well as acetabular hypertrophy. A triple phase bone scan showed minimally increased activity in the femoral shaft, at the tip of the femoral component due to stress reaction of the area. The patient was determined as a suitable candidate for an elective revisional THA.

**Figure 1.**
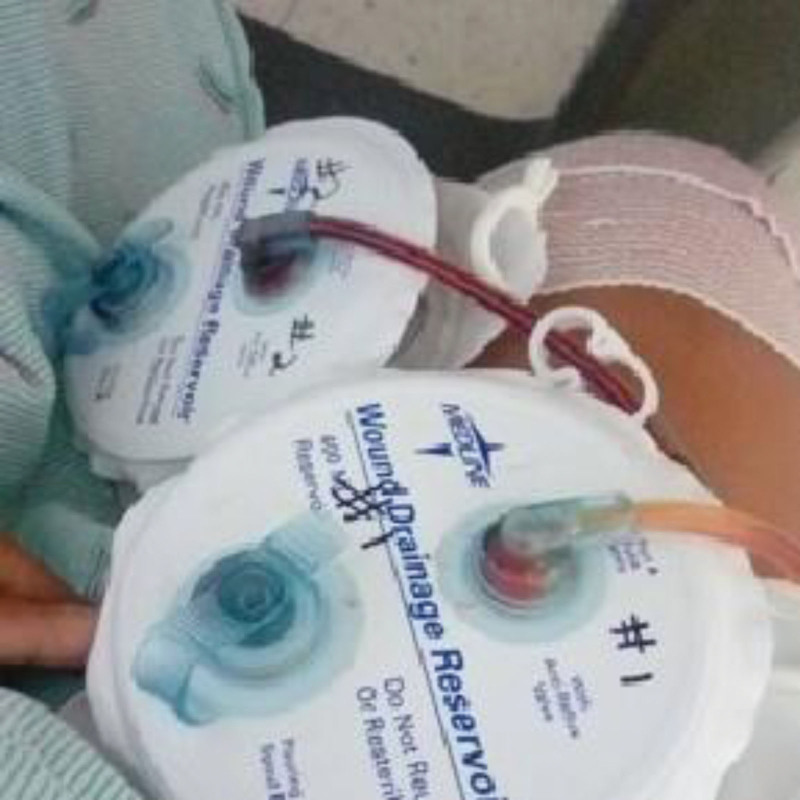
WoundVAC with percutaneous drains.

Pre-operative care included a comprehensive review of systems, diet and exercise to promote weight loss and optimize blood pressure control from 157/110 to 134/81. The patient was able to reduce her weight by 13% over 11 months—with her BMI reducing to 36 kg/m^2^ from 38 kg/m^2^ during the initial consult. The procedure was performed at Humboldt Park Health, a community hospital, by a senior orthopedic surgeon with 20 years of experience. Preoperative antibiotics were given intravenously, and an anterolateral surgical approach was chosen, with a 27 cm longitudinal skin incision made over the central portion of the right hip, followed by sharp dissection through the subcutaneous tissue down to the tensor fascia lata, which was preserved and retracted. The underlying gluteus medius and minimus were split and retracted, exposing the joint capsule. The capsule and scar tissue were removed to mobilize the joint and expose the prosthesis. The acetabulum was reamed until the bleeding bone was exposed. Then the femoral and acetabular articulations were removed. The surgical site was irrigated with antibiotic pulse lavage. The new acetabulum and femoral prosthesis were implanted. Reduction of the prosthesis was done and checked for stability with additional cerclage cables to stabilize the proximal femur. The gluteus minimus and medius were approximated and anchored with #1 polydioxanone sutures to the trochanter. After the surgical procedure, the WoundVAC and the percutaneous drain were applied outside the tensor fascia lata to reduce seroma and hematoma formation (Fig. 1). The implants used were Zimmer, Trilogy/TM, Acetabulum, Cementless (56 mm); Zimmer, Trilogy/TM, Acetabulum, Liner (40 mm); Zimmer, Femoral Head (40/+10.5 mm); Zimmer, Femoral Stem, TM (13 mm); Cerclage Cables. Estimated blood loss for this procedure was 750 mL, for which the patient received 1 unit of blood transfusion. Postoperative pain control, antibiotics and DVT prophylaxis were given. On post-op day 3, an irrigation and debridement with delayed primary wound closure was performed under sterile conditions. Upon removal of the WoundVAC sponge, subcutaneous tissue was noted to be filling the defect well. Irrigation with pulsatile lavage and culture was performed at the fat layer and soft tissues around the hip, followed by removal of the drains. Surrounding muscle, fascia were reapproximated with #1 polydioxanone sutures and skin was closed with #2 and 2-0 nylon suture and staples. Sterile dressing was applied.

**Figure 2.**
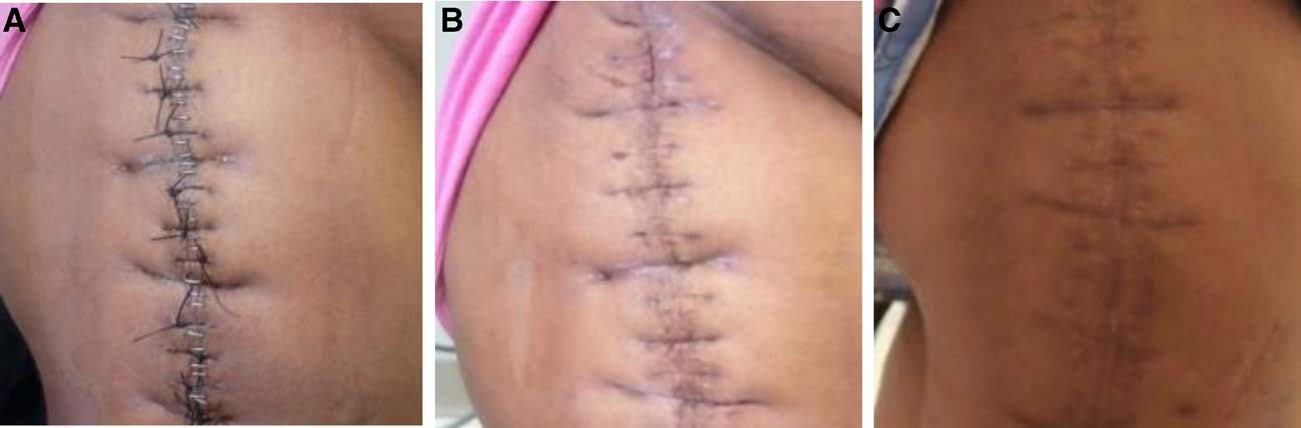
(A) Four wk post-operative wound site—The wound is healing well with no signs of infection. The tensor fascia lata was closed with #1 PDS and the skin was closed with #2 and 2-0 nylon sutures and skin staples on post-op d 3. (B) Four wk post-operative after removal of staples ad skin sutures from wound site—Surgical staples and skin sutures were removed from the surgical incision and the wound has healed. (C) Six wk post-operative follow-up of wound site—The skin is healed with no signs of local inflammation or infection. PDS = polydioxanone sutures.

On all postoperative assessment, the patient had intact sensation, capillary refill within normal range, and adequate pain control. The patient was started on physiotherapy rehabilitation. On follow-up visits, the wound demonstrated adequate healing without any signs of infection. Sutures and staples were removed 4 weeks post-op (Fig. 2A and B). Upon palpation there was no edema, effusions, temperature changes, tenderness. Clinical inspection revealed symmetrical alignment of the pelvis and hips. Range of motion testing revealed restriction beyond 80 degrees upon flexion and beyond 5 degrees of adduction and 10 degree of abduction. The surgical site was noted to be healed at 6 weeks post-op (Fig. 2C). The skin was normal in color, texture, moisture, temperature, mobility and turgor. Patient was recommended to start weight bearing as tolerated and start utilizing a cane instead of the walker. The patient continued to do well to date, without exacerbations.

## 3. Clinical discussion

We discuss an interesting case highlighting the potential role of fat migration in improved wound healing aided by NPWT following a revisional THA in a patient with multiple comorbidities, putting her at a higher risk of postoperative complications and significantly delayed healing. Possible complications include wound healing failure, fascial dehiscence, and surgical site infections in the setting of obesity.^[3,4]^ A BMI of over 30 kg/m^2^ is the clinical definition of obesity, which classified our patient in this category. In the literature, the correlation between obesity and poor tissue hypoperfusion has been documented as a predisposing risk factor for poor wound healing. With an increase in fat cells disproportionate to the blood flow of the area, a relative lack of tissue oxygenation would delay wound healing.^[4]^ Moreover, fat tissue has been correlated with a surge of pro-inflammatory cytokines that prolong the inflammatory stage of wound healing.^[6]^

BMI > 30, blood loss of >300 mL, and blood transfusions are known to be high infection risk factors.^[7]^ Despite pre-operative improvements in weight and blood pressure, our patient was still at a risk of developing surgical site infections and longer healing time. This patient case profile put her at a higher risk of developing postoperative complications associated with wound healing, so NPWT was chosen for wound closure. NPWT refers to the application of subatmospheric pressure to a sealed wound via tube drains attached to a suction pump to promote increased blood flow to the site, angiogenesis, a decreased wound surface area and reduce the risk of contamination.^[7,8]^ NPWT has been shown to increase cell migration as well as proliferation to aid in wound closure microscopically.^[9]^

In addition to this, we explored the concept of preadipocyte migration as a mechanism that may have improved wound healing. Studies have shown that preadipocytes, which are highly migratory fibroblast-like stem cells, facilitate wound healing.^[10]^ Previous studies on transgenic mice have shown this effect can be explained by the role of adiponectin, which is a potent mediator of wound healing through re-epithelialization and angiogenesis.^[11]^ In addition, adiponectin promotes preadipocyte proliferation and migration, serving as a potential therapeutic approach to treating surgical wounds in complex cases such as this patient.^[12]^ Obesity correlates with markedly decreased levels of adiponectin, which could impair preadipocyte migration.^[13]^ However, exercise and weight loss increase the level of circulating adiponectin which could explain the success of preadipocyte migration in this case profile.^[14,15]^

One of the limitations in our case report include not having a method of quantification for preadipocyte migration. However, future surgical cases could utilize ultrasounds as a way to measure the depth of the subcutaneous tissue layer and act to quantify the level of fat migration that has occurred. In the future, large scale, multicenter studies exploring the potential benefits of NPWT with an emphasis on preadipocyte migration, can provide valuable evidence in this matter. Moreover, this case prompts the need to analyze the rate of wound healing and length of hospital stay after THA in patients with NPWT in comparison with control groups. NPWT with preadipocyte migration is a promising step toward development of innovative therapy for morbidly obese patients undergoing orthopedic surgeries, leading to better patient outcomes.

## 4. Conclusion

Obesity, large incision size and blood loss are risk factors for wound healing failure and formed the rationale behind the application of NPWT in this patient. This case highlights the possible role of preadipocyte migration as a complement to NPWT in wound healing. Further research on larger cohorts can help better understand the impact of weight loss on preadipocyte migration and the role preadipocyte migration plays during wound healing in complex orthopedic surgeries with the use of NPWT.

## Author contributions

**Conceptualization:** Zoya Morani, Aashna Mehta, Fnu Parul, Helen Huang.

**Data curation:** Zoya Morani, Aashna Mehta, Fnu Parul, Helen Huang.

**Formal analysis:** Zoya Morani, Aashna Mehta.

**Funding acquisition:** Zoya Morani, Aashna Mehta.

**Investigation:** Zoya Morani, Aashna Mehta.

**Methodology:** Zoya Morani, Aashna Mehta, Helen Huang.

**Project administration:** Zoya Morani, Aashna Mehta.

**Resources:** Zoya Morani, Aashna Mehta.

**Software:** Zoya Morani, Aashna Mehta.

**Supervision:** Zoya Morani, Aashna Mehta.

**Validation:** Zoya Morani, Aashna Mehta.

**Visualization:** Zoya Morani, Aashna Mehta.

**Writing – original draft:** Zoya Morani, Aashna Mehta, Fnu Parul, Helen Huang, Mohammad Raza Khan, Nialish Javaid, Rachael Suzanne Ninan, Andrew Awuah Wireko, Luqman Muhammed, Fatima Aman Siddiqui.

**Writing – review & editing:** Zoya Morani, Aashna Mehta, Fnu Parul, Helen Huang, Mohammad Raza Khan, Nialish Javaid, Rachael Suzanne Ninan, Andrew Awuah Wireko, Luqman Muhammed, Fatima Aman Siddiqui.
